# Influenza A virus outbreak in Police Training School, Nazafgarh, Delhi 2009

**Published:** 2010-12

**Authors:** D.K. Raut, Saudan Singh, Neelam Roy, Deepthi Nair, Rinku Sharma

**Affiliations:** * Department of Community Medicine & VM Medical College & Safdarjung Hospital, New Delhi 110 029, India; ** Department of Microbiology VM Medical College & Safdarjung Hospital, New Delhi 110 029, India

Sir,

WHO declared pandemic Influenza Phase 6 on June 11, 2009, because of escalating outbreaks of novel H1N1 virus in several countries of more than two WHO regions^1^. In July 2009, nodal officer of Integrated Disease Surveillance Project (IDSP), South-West district, Delhi, informed us about the sudden onset of influenza like illness (ILI) among the boarders of Police Training School (PTS), Jharoda Kalan, Delhi. We investigated this outbreak to identify disease, mode of transmission and suggest control measures.

The suspected case was defined as a person with acute onset of fever, cough and sore throat with or without diarrhoea, headache, and body ache starting from July 2, 2009 onwards. Cases were searched from tent to tent and were interviewed by pre-formed proforma to collect information on travel history, contact with similar cases before onset of disease and clinical examination was also done. Environmental conditions such as lighting, ventilation, overcrowding, sanitation and humidity in the tents were also examined. Blood samples (5 ml) were collected for viral serology and blood cultures, from suspected cases having recent onset of fever. Respiratory samples (nasopharyngeal and throat swabs) were also collected and immediately plated on viral transport medium and transported under proper cold chain to National Institute of Communicable Diseases (now National Centre for Disease Control), Delhi for confirmation of Influenza A H1N1 by TaqMan-based RT-PCR tests^2^,^3^. Throat and nasopharyngeal swabs were also collected for pyogenic/bacterial and fungal growth.

Of the 1600 male trainees living in PTS in about 54 tents, 74 (4.6%) cases were found. A sudden rise in influenza like illness started on 2^nd^ July 2009 following the spells of heavy rain. Higher ILI incidence during July 2 to 10, 2009, as compared to preceding daily average rates (7 vs. 2 patient visits/24 h) were noticed confirming outbreak. Frequency of cases reached peak on 7^th^ July 2009 then declined to pre outbreak level on 17^th^ July 2009. Two samples (20%) out of five were positive for *Influenza* A. However, all the samples were negative for *Influenza A* H1N1. All cases were male (PTS has only male students), between 20 to 25 yr with mean age 22.2 ± 5.04 yr. Maximum cases were of 21 (36.4%) yr. No death was reported. Symptoms associated with fever were sore throat 37(50.8%), myalgia 23(30.2%), headache and body ache 11(15%), and few other symptoms of loose motions, generalized weakness and dysphagia. The sensitivity of clinical definitions for ILI (defined as fever and cough) ranged from 63 to 78 per cent; and specificity from 55 to 71 per cent, when compared to diagnosis by viral culture^4^,^5^. Incubation period of Influenza A in the present outbreak was short (few hours to three days). In previous studies, the typical incubation period for influenza of 1 to 4 days, with an average of 2 days has been reported^6^. Almost all the tents had cases with an attack rate of 3.6 to 16.3 per cent trainees per tent. In closed setting *e.g.,* ships and military base attack rates had been reported as high as 37-45 per cent^7^,^8^.

Overcrowding, inadequacy of natural ventilation and lighting were present in all tents. Heavy rains before outbreak leading to humid conditions in tents facilitated person to person transmission of *Influenza A* may be by droplet infection/nuclei created by sneezing, coughing and talking.

Control measures were employed from the day of outbreak investigation *e.g.,* awareness among trainees about the present outbreak, precautionary measures (hand hygiene, cough etiquettes), case finding and placing patients on sick leave to prevent future occurrences.
